# Transcriptome profiling reveals differential gene expression in proanthocyanidin biosynthesis associated with red/green skin color mutant of pear (*Pyrus communis* L.)

**DOI:** 10.3389/fpls.2015.00795

**Published:** 2015-09-30

**Authors:** Yanan Yang, Gaifang Yao, Wenquan Yue, Shaoling Zhang, Jun Wu

**Affiliations:** ^1^State Key Laboratory of Crop Genetics and Germplasm Enhancement, Centre of Pear Engineering Technology Research, Nanjing Agricultural UniversityNanjing, China; ^2^Pear Fruit Research Centre, Changli Institute of Pomology, Hebei Academy of Agriculture and Forestry SciencesChangli, China

**Keywords:** “Starkrimson”(*Pyrus communis* L.), red/green skin color mutant, differentially expressed gene (DEG), proanthocyandin biosynthesis, *LAR* (leucoanthocyanidin reductase), *ANR* (anthocyanidin reductase)

## Abstract

Anthocyanin concentration is the key determinant for red skin color in pear fruit. However, the molecular basis for development of red skin is complicated and has not been well-understood thus far. “Starkrimson” (*Pyrus communis* L.), an introduced red pear cultivated in the north of China and its green mutant provides a desirable red/green pair for identification of candidate genes involved in color variation. Here, we sequenced and annotated the transcriptome for the red/green color mutant at three stages of development using Illumina RNA-seq technology. The total number of mapped reads ranged from 26 to 46 million in six libraries. About 70.11–71.95% of clean reads could be mapped to the reference genome. Compared with green colored fruit, a total of 2230 differentially expressed genes (DEGs) were identified in red fruit. Gene Ontology (GO) terms were defined for 4886 differential transcripts involved in 15 Kyoto Encyclopedia of Genes and Genomes (KEGG) pathways. Three DEGs were identified as candidate genes in the flavonoid pathway, *LAR, ANR*, and *C3H*. Tellingly, higher expression was found for genes encoding *ANR* and *LAR* in the green color mutant, promoting the proanthocyanidin (PA) pathway and leading to lower anthocyanin. MYB-binding cis-motifs were identified in the promoter region of *LAR* and *ANR*. Based on these findings, we speculate that the regulation of PA biosynthesis might be a key factor for this red/green color mutant. Besides the known *MYB* and *MADS* transcription families, two new families, *AP2* and *WRKY*, were identified as having high correlation with anthocyanin biosynthesis in red skinned pear. In addition, qRT-PCR was used to confirm the transcriptome results for 17 DEGs, high correlation of gene expression, further proved that *AP2* and *WARK* regulated the anthocyanin biosynthesis in red skinned “Starkrimson,” and *ANR* and *LAR* promote PA biosynthesis and contribute to the green skinned variant. This study can serve as a valuable new resource laying a solid foundation for functional gene identification in the anthocyanin pathway of red-skinned pear and provide a good reference for relevant research on molecular mechanisms of color variation in other pear species.

## Introduction

Red coloration is an appealing feature in many flowers, fruits, and other plant tissues and is associated with anthocyanin accumulation. Anthocyanins also play an important role in plant disease resistance and protection against ultraviolet radiation (Bieza and Lois, 2001), and have been generally considered to have antioxidant capability (Veeriah et al., 2006), leading to the prevention of neuronal and cardiovascular illnesses, cancer, and diabetes in humans (Konczak and Zhang, 2004).

Pear fruit (*Pyrus*) is cultivated world-wide, with China ranking as the top producer of Asian pear. However, few red-skinned pears are cultivated and sold in China. Comparatively more red-skinned cultivars are found in European pear (*Pyrus communis*), which provides a good resource for red skin color and the possibility for color improvement of Asian pear. It is well-known that flavonoid biosynthesis, such as the anthocyanins in pear skins, is genetically catalyzed by structural genes encoding key enzymes (Holton and Cornish, 1995; Davies and Schwinn, 2003). In addition, these structural genes involved in anthocyanin biosynthesis have been reported to be controlled by a specific transcriptional complex, first reported in the model plant *Arabidopsis thaliana* (Zhang et al., 2003; Rowan et al., 2009). Subsequently, grapevine (Hichri et al., 2010), apple (Espley et al., 2007; Lin-Wang et al., 2011; Xie et al., 2012) Chinese bayberry (Liu et al., 2013c) have all been reported to have the transcription factors *MYB, bHLH*, and *WD40* coordinated with each other to promote anthocyanin accumulation. Recently, it has also been reported that *NAC* transcription factors activate anthocyanin biosynthesis in blood-fleshed peach (Zhou et al., 2015).

As for anthocyanin biosynthesis in other fruit species, the understanding of the molecular mechanism of anthocyanin biosynthesis in red-skinned pear has also advanced recently. The important structural genes phenylalanine ammonialyase (*PAL*), chalcone synthase (*CHS*), chalcone isomerase (*CHI*), flavanone 3-hydroxylase (*F3H*), dihydroflavonol-4-reductase (*DFR*), anthocyanidin synthase/leucoanthocyanidin dioxygenase (*ANS/LDOX*), UDP-glucose: flavonoid-3-O-glucosyltransferase (*UFGT*), and transcription factors *MYB10, bHLH*, and *WD40* have been successfully isolated in pears (Fischer et al., 2007; Feng et al., 2010; Zhang et al., 2011; Yang et al., 2013). Previous studies reported that *MYB10* plays an important role in anthocyanin biosynthesis of apples (Espley et al., 2007), strawberry (Medina-Puche et al., 2014), nectarine (Rahim et al., 2014), and other fruit trees, while in red-skinned pear, anthocyanin biosynthesis is regulated by a R2R3-MYB transcription factor *PyMYB10* (Feng et al., 2010; Zhang et al., 2011; Yu et al., 2012). However, Pierantoni et al. (2010) reported that *PcMYB10* is not directly responsible for red vs. yellow color in “Max Red Bartlett” and “Williams” pear varieties by map position, as the mutation underlying this difference maps to a different region of the pear genome. Our study on a pair of red/green pears also revealed that structural genes *PAL, CHS, CHI, F3H, DFR, ANS/LDOX*, and *UFGT* did not have mutations but were more highly expressed in the red pear. *MYB10* was only significantly more expressed at the early stage, while the expression levels of *bHLH* and *WD40* were higher at a later stage (Yang et al., 2013). These data indicated that the high expression of structural genes in the anthocyanin biosynthesis pathway led to the red skinned pear, and *MYB10* does not appear to be the key transcription factor regulating the biosynthesis of anthocyanin and determining the red/green color mutant (Yang et al., 2013). In addition, we reported differential regulation mechanisms of anthocyanin biosynthesis and coloration pattern between occidental and oriental pears by the different co-expression of *MYB10* and *bHLH33* and expressions of *WD40* (Yang et al., 2015). This all indicates that the molecular mechanisms of coloration can be different even between different varieties of the same fruit. So far, the red color trait in pear is comparatively more complex than expected, and the regulatory molecular mechanism has not been consistently concluded. Hence, the underlying genetics of anthocyanin biosynthesis and the metabolic pathway still need further exploration.

“Starkrimson” (*Pyrus communis*) is called “Early Red Doyenne Du Comice” in China, and was introduced from England to China by Changli Institute of Pomology (Hebei Academy of Agriculture and Forestry Sciences) in 2001. The fruit of “Starkrimson” has purplish red skin with the color covering the whole fruit, from the very beginning of fruit setting to maturation. The red coloration of fruit is not affected by growing conditions and show stable red color in different orchards distributed in different provinces. The green mutant of “Starkrimson” was obtained by mutagenesis with Co^60^-γ ray, and were stabilized for 5 years at very early stages of fruit development and continued into the harvesting stages (Wu et al., 2013b; Yang et al., 2013). So the color difference of the “Starkrimson” and variant strain is controlled by genetic factors, and not environmental conditions (Yang et al., 2013). The artificial induction of skin color variations offer a good opportunity to elucidate the role of genes controlling and regulating anthocyanin biosynthesis in pear fruit. In our previous study, we found the candidate gene *PyMADS18*, which might be involved in regulating anthocyanin biosynthesis, by screening the cDNA libraries of a pair of red and green pears (Wu et al., 2013b). We also cloned seven anthocyanin biosynthesis genes in red/green fruit skin mutant strains and detected no sequence differences between the color mutants, which indicated that the skin color change was not caused by a mutation of any of these genes. However, their expression levels differed, leading us to conclude that unknown genes might play an important role in formation of the red color, and new sequencing technologies provide an effective way of studying differentially expressed genes at the genome level.

High-throughput sequencing technologies can generate large amounts of sequence data cheaply and quickly, and have been applied widely to transcriptome analysis of plants and animals. The transcriptome is the complete set of expressed RNA transcripts during a developmental stage or in response to a particular physiological condition. It provides valuable information for identifying differentially expressed genes, and its impact on modern plant breeding gives it broad use and application, such as in *M. sprengeri* (Shi et al., 2014), pummelo (*Citrus grandis*) (Guo et al., 2015), grapevine (*Vitis labrusca* × *V. vinifera*) (Cheng et al., 2015), and peach (Zhou et al., 2014). The aim of transcriptome analysis is to screen a series of candidate genes associated with the traits of interest, and to lay a good foundation for further identifying gene functions and carrying out genetic improvement of the traits. The first draft genome of the pear (*Pyrus bretschneideri*) was reported recently (Wu et al., 2013a) and provides a good platform and reference for transcriptome analysis. Recently, the calyx abscission process of “Kuerlexiangli” pear (*Pyrus sinkiangensis Yu*) was reported (Qi et al., 2013) through RNA-seq analysis and identified candidate genes with highly dynamic changes in expression during the calyx abscission process. Subsequently, the analysis of surface brown spot formation in pear fruit was reported (Wang et al., 2014), exploring differentially expressed genes between russet and green pericarp offspring of a sand pear cv. “Qingxiang” × “Cuiguan” F1 population. Examination of a selected set of these categories revealed repressed expression of candidate genes for suberin, cutin, and wax biosynthesis in the russet pericarps. Also, the bud release following early defoliation of “Hosui” (*Pyrus pyrifolia*) was analyzed by RNA-seq (Zhang et al., 2015) for gene expression in pear floral buds of completely defoliated plants after harvest. These transcriptome studies provide a comprehensive molecular biology insight via the differentially expressed genes into the target traits and screening for important candidate genes.

In this study, we sequenced the transcriptome of red-skinned “Starkrimson” and its green-skinned mutant using Illumina RNA-seq technology. A set of up-regulated and down-regulated genes between red- and green-skinned pear fruits were screened and help to reveal the molecular mechanism for the color mutation from red to green. Some important candidate genes were identified related to anthocyanin synthesis and red skinned fruit of “Starkrimson.” The assembled annotated transcriptome sequences provide a valuable genomic resource to further understand the molecular basis of regulation of anthocyanin biosynthesis in pear. In addition, the results and strategy will also contribute to relevant research on molecular mechanisms of color variation in other fruit species.

## Materials and methods

### Plant materials and samples collection

The plant materials used in this study were the red-skinned pear “Starkrimson” and its green mutant, obtained from the orchard of Changli Institute of Pomology in Hebei Province during the 2012 growing season. The growth conditions of “Starkrimson” and its green mutant were described in our previous research (Wu et al., 2013b). The samples were collected at 40, 55, and 85 days after full bloom (DAFB). For these samples, 12 consistent fruits of each sample were randomly divided into three groups (for fruitlets at the early stage, 30 of each sample were selected). The fruit skin was peeled off and immediately frozen in liquid nitrogen and stored at −80°C. Part of the samples were used to measure anthocyanin content, others were used to extract RNA and for transcriptome sequencing. For red- and green-skinned pears, a total of six independent libraries were sequenced, and samples were named as red 1 and green 1 at 40 DAFB; red 2 and green 2 at 55 DAFB; and red 3 and green 3 at 85 DAFB (Table 1).

**Table 1:** **Summary of the transcriptome reads for red and green skinned fruits**.

**Samples**	**Raw reads**	**Single length (bp)**	**Pair end (Y/N)**	**Total length (bp)**	**Clear reads rate (%)**	**Mapping to genome**		**Spliced reads**	
							**Rate (%)**		**Rate (%)**
Red 1	26405278	100	Y	5281055600	88.05	35214283	71.3	11348428	24.65
Red 2	29523382	100	Y	5904676400	87.61	39567632	71.95	11627416	15.92
Red 3	28745012	100	Y	5749002400	88.12	38446058	71.6	13001771	26.18
Green 1	46456562	100	Y	9291312400	90.93	61883293	70.11	19801638	26.03
Green 2	29955395	100	Y	5991079000	91.15	40728952	71.47	14479451	29.56
Green 3	32641354	100	Y	6528270800	87.30	43777266	71.71	14931052	27.59

“*Raw reads” means the number of paired-end reads, “Spliced reads” means one read spliced-mapped to two exon reads and the rate (%) means the percentage of the spliced reads/clean reads. Red 1, red-skinned fruit at 40 DAFB; Red 2, red-skinned fruit at 55 DAFB; Red 3, red-skinned fruit at 85 DAFB; Green 1, green-skinned fruit at 40 DAFB; Green 2, green-skinned fruit at 55 DAFB; Green 3, green-skinned fruit at 85 DAFB*.

### Measurement of anthocyanin content in skins of pear

Anthocyanin extraction of the samples was done using 1 g pear fruit skins in 5 mL 1% HCl-methanol (V/V) for 24 h at 4°C with shading. After centrifugation for 20 min at 12 000 g, the upper aqueous phase was subjected to spectrophotometric quantification at 530, 620, and 650 nm using a UV–vis spectrophotometer (MAPADA UV-1800, China). The relative anthocyanin content was determined with the following formula: OD = (A_530_–A_620_)–0.1(A_650_–A_620_) (Lee and Wicher, 1991). One unit of anthocyanin content was expressed as a change of 0.1 OD (unit × 10^3^ g^−1^FW). At least three independent samples from each group were used to obtain the mean anthocyanin content.

### RNA extraction and transcriptome sequencing using illumina platform

The total RNA was extracted using Plant RNA Isolation Kit (Auto Lab), followed by RNA purification with RNeasy MiniElute Cleanup Kit (Qiagen) according to the manufacturer's instructions. RNA degradation and contamination was monitored on 1% agarose gels. RNA purity was checked using a NanoPhotometer® spectrophotometer (IMPLEN, CA, USA). RNA concentration was measured using Qubit® RNA Assay Kit in Qubit® 2.0 Fluorometer. The quality of total RNA was evaluated with an Agilent 2100 Bioanalyzer (Agilent Technologies, CA, USA). Only samples with RIN (RNA integrity number) ≥ 8 and 28S:18S RNA ≥ 1.5 were used for deep sequencing. The cDNAs were quantified with Qubit® RNA Assay Kit (Invitrogen, Foster City, CA), following the instructions of the manufacturer, with initial volume range of 0.1–4 μg. For mRNA library construction and deep sequencing, RNA samples were prepared using the TruSeq RNA Sample Preparation Kit according to the manufacturer's protocol, including polyA-mRNA purification and fragment, first strand cDNA synthesis, second strand cDNA synthesis, and repair, and adapter ligation, PCR enrichment, agarose gel purification, and library quality control. The library was sequenced using an Illumina HiSeq™ 2000 (San Diego, CA, USA) by CapitalBio Corporation (Beijing, China).

### Data analysis

Raw reads obtained by the HT-2000 were filtered to exclude low complexity reads and reads containing adaptor sequences. The resulting clean reads were assembled with Trinity (Grabherr et al., 2011), and The Gene Index Clustering Tool (TGICL) (Pertea et al., 2003) was used to optimize the original Trinity assembly result by removing sequences that could not be extended on either end. The remaining high quality sequences (clean reads) were mapped to the assembled pear genome data (Wu et al., 2013a) using Bowtie (Kanehisa et al., 2006). The assembled transcripts were also annotated using Blast2GO (Conesa et al., 2005) with GO (Ashburner et al., 2000) and KEGG (Kanehisa et al., 2008). The calculation of transcript expression was with the RPKM method (Reads Per kb per Million reads) (Mortazavi et al., 2008). Differentially expressed genes (DEG) analysis was performed using the method described by Audic and Claverie (1997) False discovery rate (FDR) (Benjamini and Yekutieli, 2001) was used to determine the *p*-value thresholds in multiple testing. Finally, genes with a *P* ≤ 0.01 and Fold Change ≥ 2 were marked significantly different between the two libraries. In addition, the transcriptomic data supporting the results of this article are available at NCBI under BioProject with accession number PRJNA290937 with SRA Study accession number SUB1036370 (https://submit.ncbi.nlm.nih.gov/subs/biosample/).

### Real-time PCR analysis

To validate the expression patterns revealed by DEG results, 17 identified genes were analyzed using quantitative real-time PCR. RT-qPCR amplification and analysis were performed with the LightCycler 480 SYBR GREEN Master (Roche, USA), according to the manufacturer's instructions. The primers used for amplifying each gene are presented in Table 2. The raw data were analyzed with LightCycler 480 Software release version 1.5.0 (Roche), and the gene expression levels were determined with the 2^−ΔΔ*T*^ algorithm by normalizing to the *Pyrus* EFα1 (EFα1, accession number AY338250) and *Pyrus* TUB-b2 (TUB-b2, accession number AB239681) (Wu et al., 2012). qRT-PCR data are technical replicates with error bars, representing means ± SE (*n* = 3). Statistical and correlation analysis was performed with *SPSS* for Windows NT (release 8.0.0).

**Table 2:** **The distribution region of clean reads mapped to the reference genome**.

**Class**	**Red 1**		**Red 2**		**Red 3**		**Green 1**		**Green 2**		**Green 3**	
	**#**	**%**	**#**	**%**	**#**	**%**	**#**	**%**	**#**	**%**	**#**	**%**
Total mapped position	46047389	100	73032011	100	49663098	100	76065118	100	48987916	100	54114474	100
Exon	25196602	54.72	31364377	42.95	27474374	55.32	43343094	56.98	27807170	56.76	30284252	55.96
Intron	1458748	3.17	3956114	5.42	1363360	2.75	2295183	3.02	1073854	2.19	1495195	2.76
Intergenic	8043611	17.47	26084104	35.72	7823593	15.75	10625203	13.97	5627441	11.49	7403975	13.68

“*Total mapped position” means the numbers and the rate (%) of the mapped to the positions of clean reads mapped to the reference genome; “Exon” means the numbers and the rate (%) of clean reads mapped to exon of the reference genome; “Intron” means the numbers and the rate (%) of clean reads mapped to the intron of the reference genome; “Intergenic” means the numbers and the rate (%) of clean reads mapped to the intergenic of the reference genome*.

## Results and discussion

### Changes of anthocyanin content in skins during fruit development

The anthocyanin content of “Starkrimson” and the green variant were measured in our previous study (Yang et al., 2013). The anthocyanin content of the red-skinned pear “Starkrimson” was significantly higher than its green mutant, from ten-fold higher at 40 DAFB to seven-fold higher at 85 DAFB, with the highest difference between the red and the green color mutant at the early fruit development stage, that is, 40 DAFB. Anthocyanin content changes in fruit development in other red pear species show similar patterns. Wang et al. (2013) reported that the concentration of anthocyanin in “Max Red Bartlett” Pear fruits increased from 9 DAFB, peaking at 45 to 55 DAFB, and then gradually decreased through 85 DAFB to the mature fruit stage. Yang et al. (2015) reported that the anthocyanin content of the occidental pear was highest at the early stage of fruit development, and then showed a tendency to drop during fruit development and maturation. Oriental pears have lower anthocyanin content than occidental pears in general, and the anthocyanin content first increased and reached maximum values at 85 DAFB and then decreased in the later development stages in different cultivars of pears. These results indicate that anthocyanin accumulation in pear fruit skin mainly appears in early developmental stages, with fruit color gradually fading in later stages.

### DEG library sequencing and mapping sequences to the reference transcriptome database

High-throughput sequencing of RNA was performed to obtain a global view of gene expression difference related to the anthocyanin biosynthesis between “Starkrimson” and its green skin mutant. The three red- and three green-skinned pear samples were used for DEG library sequencing analysis. The number of raw reads for each library ranged from 26 to 46 million. The total length of clean reads ranged from 46 to 84 million and the rate of clean reads/raw reads ranged from 87.30 to 91.15% (Table 1). The clean reads of the six libraries were mapped to the assembled pear genome reference sequence of “Dangshansuli” (Wu et al., 2013a), with a mapping rate from 70.11 to 71.95% (Table 1). Mapping region classifications included exon, intron, intergenic, and spliced. In the six libraries, the ratios of exons were the highest, from 42.95 to 56.98%, and the ratios of introns were the lowest, from 2.19 to 5.42% (Table 2). In addition, the Q30 percentages (a Q-score of 30 corresponds to an error rate of 1 per 1000) of all six libraries were above 95%, which indicated that the sequencing and RNA qualities were high, and the data obtained was reliable enough for further profile studies on gene expression.

### Identification of genes showing differential expression between red and green fruit

**Figure 1:**
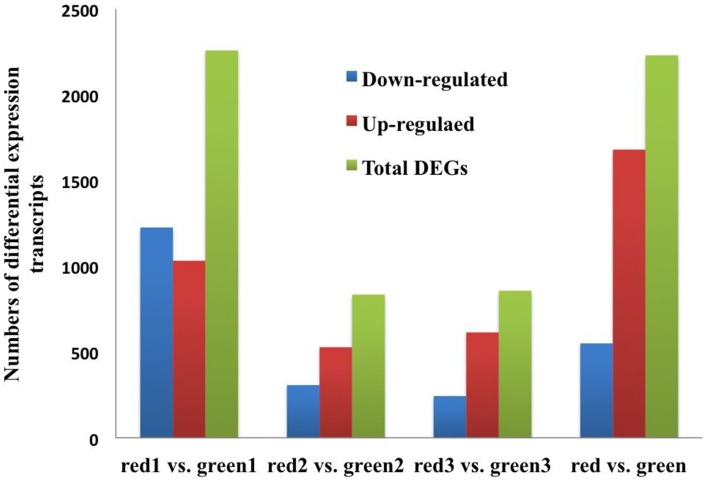
**The numbers of DEGs between red/green skin color mutant pairs**. Up-regulated (red), down-regulated (blue), and Total DEGs (green) were quantified. The results of four comparisons are shown.

**Figure 2:**
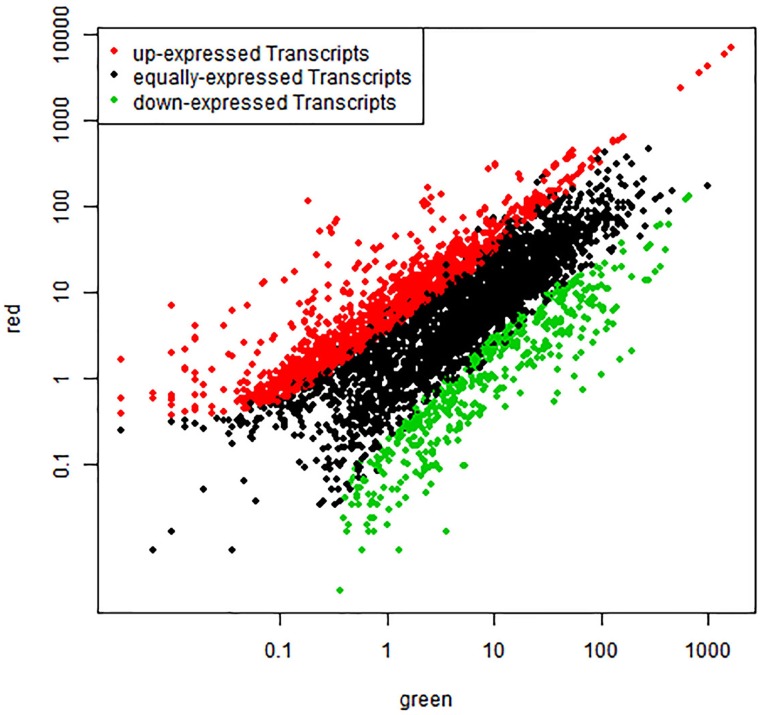
**Comparison of transcript expression between red and green fruit**. The abundance of each gene was normalized as reads per kb per Million reads (RPKM). The differentially expressed genes are shown in red and green, while black indicates genes that were not differentially expressed (not DEGs) between red and green fruit.

To compare differential expression between red and green coloration in pear fruit, we used RPKM to investigate transcript enrichment, which can eliminate the influence of differences in gene length and sequencing level. Furthermore, we employed IDEG6 to identify mRNAs showing statistically significant differences based on their relative abundance. We compared the samples from different colored fruits at the same developmental stage, so that three pairs of comparisons were implemented (Figure 1). Among these comparisons, we found that the most differentially expressed transcripts were between red 1 vs. green 1, with 1032 up-regulated unigenes and 1226 down- regulated unigenes. Simultaneously, anthocyanin content reached peak values at the early stages of fruit development. Fewer unigenes were observed between red 2 and green 2, in which only 835 unigenes were identified. Of these genes, 528 were up- regulated and 307 were down- regulated. In general, compared with green color fruit, a total of 2230 unigenes were significantly differentially expressed. Among all unigenes, 1680 up-regulated unigenes and 550 down-regulated unigenes were identified in red skinned fruits. The plots of unigenes between red and green revealed unigenes with both fold change and significance (Figure 2).

### GO classification and the enrichment analysis of differentially expressed genes (DEGs)

**Figure 3:**
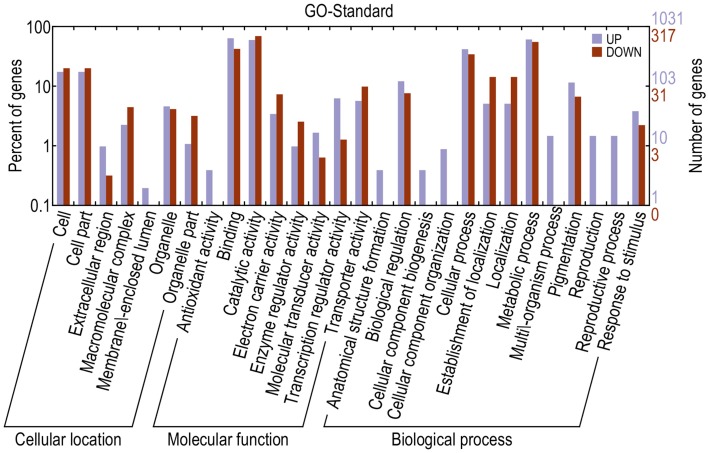
**Functional categorization of genes with significant transcriptional changes between red and green fruit**. Purple indicates up-regulated and red indicated down-regulated genes.

Gene Ontology (GO) is an international standardized gene function classification system that describes properties of genes and their products in any organism. In order to create a profile of gene expression in red-skinned pear, we used WEGO (Web Gene Ontology Annotation Plot) for gene annotation analysis. To screen the candidate genes of DEGs from the transcripts of functional annotation, we analyzed the 4886 differential transcripts at three developmental stages from a total of 174,810 transcripts in the red/green color mutant pair. The DEGs were categorized into 28 functional groups on the basis of their biological processes (Figure 3). The major subcategories were as follows: Seven subcategories for cellular location; eight subcategories for molecular function, and 13 subcategories for biological process. The DEGs in “binding,” “catalytic activity,” “metabolic process,” and “cellular process,” “cell,” “cell part,” and “pigmentation” played important roles during the pigment metabolic process. The results provided a comprehensive view for screening candidate genes related to anthocyanin biosynthesis and metabolic process of red/green color pear fruit. Taking into account that anthocyanin content of red color fruit at different developmental stages is higher than in the green mutant, we searched for DEGs at the three developmental stages, and found that among all of differentially expressed genes, there are seven up-regulated and three down-regulated genes with significant differences at all three stages (Table 3). In the up-regulated unigenes, Pbr013927.1 is an *AP2/ERF* domain sequence-specific DNA binding transcription factor and Pbr039146.1 and Pbr008092.1 are transporters from proton-dependent oligopeptide transporter and sugar/inositol transporter families. In addition, there were three unigenes encoding enzymes, oxoglutarate/iron-dependent dioxygenase (Pbr021636.1), alcohol dehydrogenase (Pbr021220.1), glutathione S-transferase (GST) (Pbr012649.1), and a hypothetical protein (Pbr039734.1) with unknown function also up-regulated. The three down-regulated unigenes were protein kinase activity (Pbr041919.1), RNA recognition motif domain protein (Pbr041849.1) and mitochondrial carrier protein (Pbr019417.1).

**Table 3:** **Distributions of all unigenes and differentially expressed genes (DEGs) in KEGG database classification**.

**Locus**	**Reference**	**Mark**	**Blast result**
XLOC_029515	Pbr039734.1	Up	Hypothetical protein
XLOC_029062	Pbr039146.1	Up	Proton-dependent oligopeptide transporter family(hypothetical protein)
XLOC_016104	Pbr021636.1	Up	Oxoglutarate/iron-dependent dioxygenase
XLOC_015900	Pbr021220.1	Up	Alcohol dehydrogenase
XLOC_010359	Pbr013927.1	Up	AP2/ERF domain sequence-specific DNA binding transcription factor activity
XLOC_009288	Pbr012649.1	Up	Glutathione S-transferase (GST)
XLOC_005882	Pbr008092.1	Up	Sugar/inositol transporter (hypothetical protein)
XLOC_031133	Pbr041919.1	Down	Protein kinase activity At5g41260
XLOC_031072	Pbr041849.1	Down	RNA recognition motif domain
XLOC_014579	Pbr019417.1	Down	Mitochondrial carrier protein

“*Up” means genes up-regulated in the red skinned pear relative to the green skinned pear in different developmental stages, and “Down” means genes down-regulated in the red skinned pear relative to the green skinned pear in different development stages*.

### Kyoto encyclopedia of genes and genomes (KEGG) enrichment analysis of DEGs

KEGG analysis provides information and further understanding on how “Starkrimson” pear regulates its biological functions and synthesizes secondary metabolites, including anthocyanin, at the molecular level. Usually, unigenes in the same pathway cooperate with each other. In this study, there were 42,684 unigenes that mapped to 248 KEGG pathways using Blast X homology search. Furthermore, there were 783 DEGs mapped in 101 KEGG pathways, including metabolism (595 DEGs), genetic information processing (68 DEGs), environmental information processing (58 DEGs), cellular processes (36 DEGs), and human diseases (26 DEGs) (Table 4). Among them, 12 pathways were significantly enriched with more than 15 DEGs (*Q* ≤ 0.05) (Table 5). In the metabolism category, the most abundant DEGs were found in the amino acid metabolism sub-category (PATH:ko00270, PATH:ko00330, PATH:ko00250; PATH:ko00071), followed by carbohydrate metabolism (PATH:ko00010), lipid metabolism (PATH:ko00561; BR:ko01004), and metabolism of other amino acids (PATH:ko00982; PATH:ko00980) (Table 4). Five hundred ninety five DEGs were related to catalysis of metabolism processes or generation of energy for primary and secondary metabolite production. We also found 3 DEGs in the secondary metabolite pathway of flavonoid biosynthesis (PATH:ko00941): p-coumarate 3-hydroxylase (*C3H*), Anthocyanidin Reductase (*ANR*), and leucoanthocyanidin reductase (*LAR*). In addition, the genetic information processing category included transcription (21 DEGs) (BR:ko03000) (Table 6), folding, sorting, and degradation (31 DEGs), replication, and repair (16 DEGs). The DEGs in this category mainly function in ensuring correct transcription and translation processes, and the transcription factors indirectly involved in fruit development transcription regulation and chromosome (BR:ko03036) determine metabolism, cellular processes, and environmental information processing.

**Table 4:** **List of the differentially expressed genes between red and green skin fruits**.

**Category**	**Sub-category**	**All genes with pathway annotation**	**DEGs with pathway annotation**
**METABOLISM**			
	Amino acid metabolism	2646	128
	Biosynthesis of other secondary metabolites	532	10
	Carbohydrate metabolism	3530	118
	Energy metabolism	3705	62
	Glycan biosynthesis and metabolism	1622	19
	Lipid metabolism	2097	107
	Metabolism of cofactors and vitamins	944	24
	Metabolism of other amino acids	690	53
	Metabolism of terpenoids and polyketides	669	10
	Nucleotide metabolism	1098	12
	Xenobiotics biodegradation and metabolism	594	52
	Total	18,127	595
**CELLULAR PROCESSES**			
	Cell communication	306	0
	Cell growth and death	1361	11
	Cell motility	282	2
	Transport and catabolism	1284	23
	Total	3333	36
**ENVIRONMENTAL INFORMATION PROCESSING**			
	Membrane transport	352	0
	Signal transduction	1760	42
	Signaling molecules and interaction	321	16
	Total	2424	58
**GENETIC INFORMATION PROCESSING**			
	Folding, sorting, and degradation	4408	31
	Replication and repair	3679	16
	Transcription	7278	21
	Total	15,365	68
**HUMAN DISEASES**			
	Cancers	879	0
	Cardiovascular diseases	67	0
	Immune system diseases	170	3
	Infectious diseases	1134	13
	Metabolic diseases	132	8
	Neurodegenerative diseases	1053	2
	Total	3435	26
Total unigenes		42,684	783

**Table 5:** **List of the important KEGG pathways, with more than 15 differentially expressed genes between red and green fruits**.

**Pathway ID**	**KEGG pathway**	**Number of DEGs**
PATH:ko04075	Plant hormone signal transduction	29
PATH:ko00270	Cysteine and methionine metabolism	28
PATH:ko00010	Glycolysis/Gluconeogenesis	25
PATH:ko00330	Arginine and proline metabolism	23
PATH:ko00071	Fatty acid metabolism	22
PATH:ko00561	Glycerolipid metabolism	20
BR:ko01004	Lipid biosynthesis proteins	18
PATH:ko00982	Drug metabolism—cytochrome P450	18
PATH:ko00980	Metabolism of xenobiotics by cytochrome P450	18
BR:ko03000	Transcription factors	18
PATH:ko00250	Alanine, aspartate, and glutamate metabolism	16
BR:ko03036	Chromosome	16

**Table 6:** **List of the differentially expressed transcription factors between red and green fruit**.

**Transcript**	**Gene ID**	**Marker**	**Interpro**
TCONS_00035591:MYBP	Pbr004276.1	Up^**^	MYB6-like
TCONS_00035592:MYBP	Pbr012624.1	Up^**^	MYB32 like
TCONS_00035850:MYBP			
TCONS_00048310:MYBP	Pbr016625.1	Up^*^	MYB
TCONS_00048376:MYBP			
TCONS_00011876:MYBP			
TCONS_00050590:MYBP	Pbr017533.1	Up^**^	MYB19
TCONS_00072401:MYBP	Pbr025360.1	Up^***^	myb-related protein MYB4-like
TCONS_00087538:MYBP	Pbr030553.1	Up^*^	myb-related protein MYB4-like
TCONS_00095803:MYBP	Pbr033541.1	Up^**^	MYB91
TCONS_00095804:MYBP			
TCONS_00095805:MYBP			
TCONS_00095827:MYBP			
TCONS_00119933:MYBP	Pbr042924.1	Up^***^	MYB90-like
TCONS_00119934:MYBP			
TCONS_00101932:EREBP	Pbr035788.1	Up^*^	AP2
TCONS_00046093:WRKY33	Pbr015939.1	Up^**^	Probable WRKY9/33
TCONS_00007268:K09264	Pbr002427.2	Up^**^	MADS15/AG-like MADS-box protein
TCONS_00087310:EREBP	Pbr030451.1	Up^***^	AP2; ethylene response factor 3
TCONS_00046683:EREBP	Pbr016185.1	Up^***^	AP2; pathogenesis-related genes transcriptional activator PTI5-like
TCONS_00064831:EREBP	Pbr022708.1	Up^***^	Ethylene-responsive transcription factor ERF073-like
TCONS_00039802:EREBP	Pbr013927.1	Up^***^	Ethylene-responsive transcription factor ERF027-like
TCONS_00107554:EREBP	Pbr037846.1	Up^**^	Ethylene-responsive transcription factor ERF113-like
TCONS_00025917:WRKY	Pbr009294.1	Up^**^	Probable WRKY17
TCONS_00076872:WRKY	Pbr026903.1	Up^**^	Probable WRKY51
TCONS_00013757:WRKY	Pbr004885.1	Up^**^	Probable WRKY40
TCONS_00105776: bHLH	Pbr037113.1	Up^***^	MYC2-like; bHLH
TCONS_00119139: bHLH	Pbr041849.1	Down^***^	PRE6-like/PRE1; bHLH

* Indicated that significant difference level p < 0.05;

** Indicated that significant difference level p < 0.01;

*** *Indicated that significant difference level p < 0.001*.

### Genes involved in flavonoid biosynthesis, metabolism, and transportation

**Figure 4:**
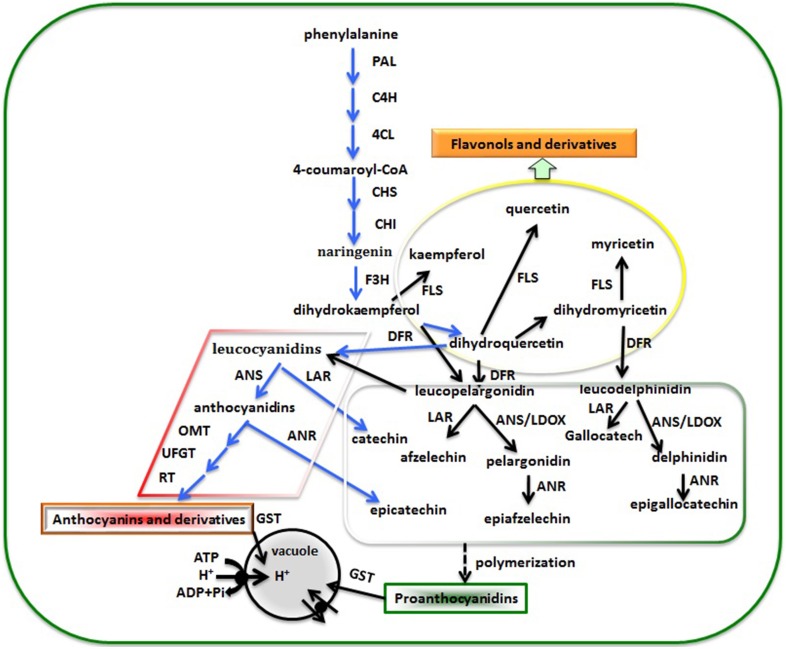
**The flavonoid biosynthesis pathway leading to PA production in pear fruit**. *PAL*, phenylalanine ammonialyase; *C4H*, cinnamate 4-hydroxylase; *4CL*, 4-coumarate coenzyme A ligase; *CHS*, chalcone synthase, *CHI*, chalcone isomerase; *F3H*, flavanone 3-hydroxylase; *FLS*, flavonol synthase; *DFR*, dihydroflavonol-4-reductase; *LAR*, leucoanthocyanidin reductase; *ANS/LDOX*, anthocyanidin synthase/leucoanthocyanidin dioxygenase; *ANR*, anthocyanidin reductase; *OMT*, O-methyltransferases; *UFGT*, UDP-glucose: flavonoid-3-O-glucosyltransferase; *RT*, rhamnosyltransferase; *GST*, glutathione S- transferase. The blue arrow indicates a process detected between red and green skinned pear, the black arrow was clarified in other plant. In addition, the rhombus shape indicates anthocyanin accumulation, the rectangle shape proanthocyanidin biosynthesis, and the ellipse shape flavonoid biosynthesis.

Flavonoid biosynthesis is a dynamic and complex processes catalyzed by a series of enzymes. Flavonoids are a diverse group of plant secondary metabolites with various biological functions that play important roles during plant development. Proanthocyanidins (PAs, also known as condensed tannins) are components of metabolites synthesized through the general flavonoid biosynthesis pathway (Figure 4). In a previous study, it was reported that leucoanthocyanidin reductase (*LAR*), anthocyanidin synthase (*ANS*; also called leucoanthocyanidin dioxygenase, *LDOX*), and anthocyanidin reductase (*ANR*; in Arabidopsis, the product of the BANYULS gene) were the three principal enzymes for flavan-3-ols biosynthesis. The synthesis of PAs and anthocyanins share common steps leading to flavan- 3,4-diols (such as leucoanthocyanidin), which can be converted to catechin (2,3-trans-flavan-3-ol) by *LAR* (Tanner et al., 2003) or to anthocyanidin by *ANS* (Abrahams et al., 2003). Anthocyanidin then either serves as the substrate for the synthesis of epicatechin (2,3-cis-flavan-3-ol) by *ANR* (Xie et al., 2003) or can otherwise be converted to anthocyanin by glycosylation (Schijlen et al., 2004). Recently, it was proven that transgenic tobacco overexpressing *TcLAR* had decreased amounts of anthocyanidins and increased PAs. Overexpressing *TcLAR* in an *Arabidopsis ldox* mutant also resulted in elevated synthesis of not only catechin but also epicatechin (Liu et al., 2013a,b). In strawberry, it was demonstrated that redirection of the anthocyanin pathway to flavan-3-ols was observed by the down-regulation of an anthocyanin glucosyltransferase in ripening strawberry fruit (Griesser et al., 2008), while the down-regulation of anthocyanin reductase (*ANR*) induced a redirection of the proanthocyanidin pathway, leading to premature, and ectopic anthocyanin biosynthesis via enzymatic glycosylation as the alternative pathway (Fischer et al., 2014). In our study, *ANR* showed relatively high expression levels in green color mutant of “Starkrimson” pear, leading to unstable anthocyanidin through *ANS* catalysis. This could not be converted to stable colored anthocyanin by 3,5-glycoside flavonoid transferase (*UFGT*), and thus anthocyanin did not accumulate in the pear fruit. Therefore, high *ANR* expression might be a main reason leading to the skin color change from red to green in this mutant. However, further study is needed to verify gene function and reveal the regulatory mechanism underlying the phenomenon. In addition, we detected significantly expressed p-coumarate 3-hydroxylase (*C3H*), which is in the flavonoid biosynthesis pathway. In a previous study, *C3H* was found to be a key gene playing an important role in lignin biosynthesis metabolism. Dardick et al. (2010) reported that p-coumarate 3-hydroxylase is involved in stone formation in peach fruit by microarray studies using a developmental series from young fruits. Recently, Xue et al. (2014) reported synthesis and codon-optimiztion of an *Arabidopsis thaliana ref8* gene encoding a *C3H* for enhanced expression in *Synechocystis*. This heterologous pathway enabled *Synechocystis* to produce caffeic acid. As this is the first report of a *C3H* being related to flavonoid metabolism in pear, the gene function still needs further study.

GST is a family of multifunctional enzymes catalyzing detoxification reactions, and are also involved in endogenous metabolism, including functioning as *GSH*-dependent isomerases, non-catalytically acting as flavonoid-binding proteins, stress signaling proteins, and regulators of apoptosis (Jain et al., 2010). In previous studies, there have been many related reports showing GST encoded enzymes involved with anthocyanin transportation from cytoplasm to vacuole. Anthocyanin synthesis is in the cytoplasm, and is then transported to the vacuole and stored, displaying different colors with different ion concentrations and pH conditions in the vacuole (Tanaka et al., 2008). Until now, it has been confirmed that some members of *GST* are involved in anthocyanin transportation in Arabidopsis (Kitamura et al., 2004), petunia (Alfenito et al., 1998), carnation (Larsen et al., 2003), corn (Marrs et al., 1995), and grapes (Conn et al., 2008). In this study, we detected *GST* (Pbr012649.1) to be up-regulated in red-skinned “Starkrimson.” However, little is known about *GST* genes related to anthocyanin transportation in pear, the full transport mechanisms still need further investigation.

### Genes encoding transcription factors

The regulation of gene expression at the transcription level has a profound role in the control of many biological processes. Transcription factors are the key switches for secondary metabolite gene regulation. Between the red and green color mutant, 21 DEGs were identified as transcription factors from the 595 DEGs annotated into metabolite sub-category of the KEGG pathway in this study (Table 6). These were annotated as *MYB, AP2, WRKY*, and *MADS* transcription factors. Among the group of transcription factors, we identified eight genes belonging to the *MYB* family of transcription factors. Previous studies have mostly focused on the superfamily of *MYB* TFs, which has been shown to be involved in the control of many biological processes, including anthocyanin biosynthesis (Takos et al., 2006), for example, a R2R3 MYB transcription factor associated with regulation of the anthocyanin biosynthetic pathway in Rosaceae (Lin-Wang et al., 2010). *MYB90/PAP2, MYB6, MYB10, MYB1, MYBA*, and *MYB19* have been reported to be involved in the regulation of anthocyanin synthesis (Borevitz et al., 2000; Takos et al., 2006; Ban et al., 2007; Gonzalez et al., 2008; Yamagishi et al., 2010; Telias et al., 2011; Zhang et al., 2012). In apple (*Malus* × *domestica*), expression of *MYB10* showed a strong correlation with anthocyanin content during fruit development of red-fleshed apple “Red Field” (Espley et al., 2007). Two more apple TFs, *MYB1* and *MYBA*, were also reported to regulate genes in the anthocyanin pathway in red-skinned fruit (Ban et al., 2007). Both *MYB1* and *MYBA* share identical sequences, while *MYB10* and *MYB1* genes are located at very similar positions on linkage group 9 of the apple genetic map (Chagné et al., 2008). In strawberry (*Fragaria* × *ananassa*), *FaMYB1* plays a key role in down-regulating the biosynthesis of anthocyanins and flavonols (Aharoni et al., 2001). *DkMYB4* is involved in proanthocyanidin biosynthesis in persimmon fruit (Akagi et al., 2009), while *AtMYBL2* encodes a protein with a single MYB domain that acts as a negative regulator of anthocyanin biosynthesis in *Arabidopsis* (Matsui et al., 2008). Recently, Guo et al. (2015) identified a number of MYB transcription factors that may be involved in the carotenoid regulation in orange-pericarp mutants and wild type in pummelo (*Citrus grandis*), via transcriptomic analysis. These results are examples of MYB genes' frequent involvement in regulating anthocyanin biosynthesis. In this study, we detected eight up-regulated *MYB* TFs in red skinned “Starkrimson,” but did not include *MYB10*, corresponding to the previous deduction that *PcMYB10* is not directly responsible for red vs. yellow color in “Max Red Bartlett” and “Williams” pear (Pierantoni et al., 2010), and other key genes control the color mutant of “Starkrimson” pear and the green variant (Yang et al., 2015). In addition, anthocyanin accumulation is related to many biological processes, and as MYB TFs is a large regulatory gene family, some *MYB* genes might be involved in anthocyanin accumulation through regulation of related biological process. So, we should further verify these new *MYB* candidate genes to find one key gene controlling red coloration of pear. In addition, the promoter region (3000 bp upstream of ATG) of *LAR* and *ANR* genes were analyzed, and were found to have MYB-binding cis-motifs in the promoter region of both genes (Additional File 1). So we speculated that MYB TFs could bind to the promoter of *LAR* or *ANR* and regulate their expression, affecting fruit coloration in pears.

In previous studies on anthocyanin biosynthesis in the flavonoid pathway, genes have been found to be coordinately modulated by a conserved *MYB-bHLH-WD40* (MBW) regulatory complex. In *Arabidopsis, bHLH EGL3* and *GL3* function in both the biosynthesis of anthocyanins and the formation of trichomes and root hairs (Ramsay and Glover, 2005). Xie et al. (2012) demonstrated that *MdbHLH3* regulates LT-induced anthocyanin accumulation and fruit coloration in apple. *MdbHLH300* is down- regulated in fruits grown in a hot climate compared with a temperate climate (Lin-Wang et al., 2011). In this study, TFs *bHLH*, Pbr037113.1, and Pbr041849.1 were up- regulated and down- regulated, respectively, indicating their different regulation roles in anthocyanin biosynthesis. We also detected that a MADS-Box protein encoding the gene *MADS15* (Pbr002427.2) was up- regulated in the early stage of fruit development. Wu et al. (2013b) screened a new transcription factor *PyMADS18*, which is likely to be involved in anthocyanin accumulation and regulation of anthocyanin synthesis in early pear fruit development. In this study, the gene (Pbr013927.1) was found to belong to *AP2/ERF* domain-containing transcription factors, and was up-regulated in red-skinned fruit at all three measured developmental stages. Four genes (Pbr030451.1, Pbr016185.1, Pbr022708.1, and Pbr037846.1) were up-regulated at both early and later stages of fruit development. In addition, we also identified four significantly differentially expressed WRKY transcription factors, *WRKY9/33* (Pbr015939.1), *WRKY17* (Pbr009294.1), *WRKY51* (Pbr026903.1), and *WRKY40* (Pbr026903.1), however, the function of these genes in the coloration of pear fruit still needs further study.

### Real-time qPCR validation of differentially expressed transcripts from transcriptome analysis

**Figure 5:**
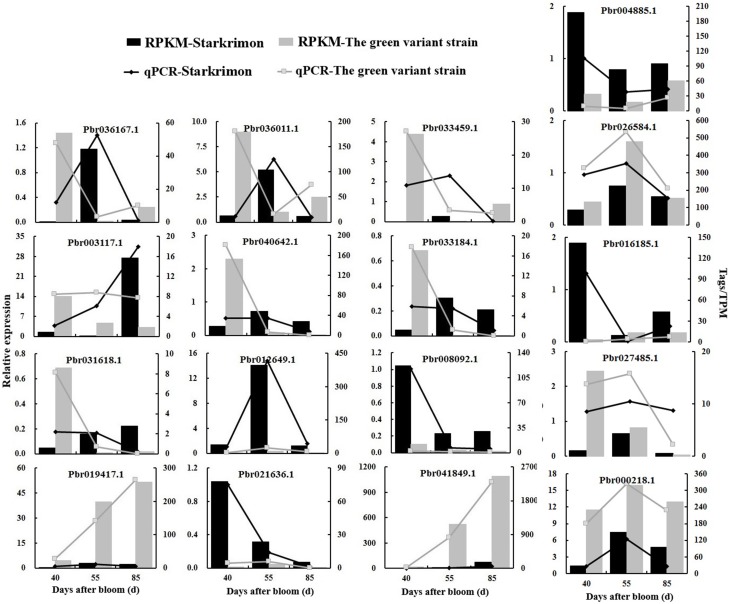
**The qRT-PCR validation of selected differential genes detected via digital transcript abundance measurements**. Development stages of pear fruit were 40, 55, and 85 DAFB. Columns show the results of digital transcript abundance measurements, the line charts show the results of qRT-PCR validation. Black indicates “Starkrimson” and gray indicates the green variant strain.

To confirm the accuracy and reproducibility of the transcriptome analysis results, 17 genes having different expression patterns were used for real-time qPCR verification and their correlation evaluated. The results showed that although the exact fold changes for the selected genes at three stages varied between digital gene expression and qRT-PCR analysis, the trend of gene expression changes detected by the two different approaches were largely consistent (Figure 5). Pearson's correlation coefficients showed that the digital transcript abundance measurements and qRT-PCR data were highly correlated (Table 7), with *R*^2^-values ranging from 0.639 (Pbr027485.1) to 0.998 (Pbr004885.1), which was in agreement with previous reports (Pan et al., 2012).

**Table 7:** **The RT-qPCR primers used for the randomly selected transcripts and the correlation analysis with digital transcript abundance measurements**.

**Transcripts**	**Genbank ID**	**Primer sequence(5′–3′)**	**Pearson's correlation coefficient**
XLOC_026873	Pbr036167.1	F-AGCAGAGCAAGAAGTGAGCG	0.965
		R-GCAAGTCGTACTGCTGTTGTG	
XLOC_026781	Pbr036011.1	F-ATGCCCTGCCAAGGTATGAC	0.985
		R-TTGCGGCTACCCTTTTCCTT	
XLOC_024882	Pbr033459.1	F-AGGGTTTTGGAGGCTTTCGT	0.822
		R-AGTGGTGCCCTTGTGTATCG	
XLOC_002279	Pbr003117.1	F-TACGAAGCCACCAAGTCCAG	0.897
		R-CCAACCCGCCTAGAAGATCC	
XLOC_030180	Pbr040642.1	F-CGCTACGATTGGGCATTGTG	0.976
		R-GATGTGGTACTTGCAGGGGT	
XLOC_024695	Pbr033184.1	F-CGGGACCGTCATCTTCAGTT	0.894
		R-CATCAAGCTGAGGACAGCCC	
XLOC_023499	Pbr031618.1	F-TTACTTGCCATGGCTTCCACA	0.905
		R-AGCGTAGGACTGTTCGCATT	
XLOC_009288	Pbr012649.1	F-TTGAGGCAGGAGAGCACAAG	0.977
		R-GGAGCACCAGAGTGTAAACCA	
XLOC_031072	Pbr041849.1	F-CAGTCAGGTACTCCAGCGAT	0.993
		R-GTACCTTGTTGGAGCGCCTAT	
XLOC_016104	Pbr021636.1	F-AACCGCTATCCCCGATCTCT	0.989
		R-CACCGGTTGTCGCTCAAAAG	
XLOC_005882	Pbr008092.1	F-CCGGTTGTGTACAGGAGGAC	0.977
		R-GCGACGACGTGAATAACTGC	
XLOC_014579	Pbr019417.1	F-GTCACTCAACCCCACGACTT	0.977
		R-CAACTAGGCCCGCTATGGAG	
XLOC_003532	Pbr004885.1	F-AACAACTGCGTGGCAATAGGA	0.998
		R-GCTTTCGTGAAATCGGGATC	
XLOC_019719	Pbr026584.1	F-TTTCTCAAACCTCCCTTACC	0.812
		R-GGATGTCGTAGCCACCAAT	
XLOC_012205	Pbr016185.1	F-GAAGGCAACTTGGGGAGGAA	0.998
		R-GGCAATAGTGAGGGGGTGAGA	
XLOC_020359	Pbr027485.1	F-AAAGTCTCCACCGAAAGCACC	0.639
		R-AGCATCTTCTTTGAAGAAACCCT	
XLOC_000077	Pbr000218.1	F-GACAGCTTGGAAATTCGCCG	0.970
		R-ATGTTACTGTGAGGGACGTCTGG	

In particular, the expression levels of screened candidate genes were in accordance with the transcriptome results. We verified candidate TFs such as *AP2* (Pbr016185.1) and *WARK* (Pbr004885.1), which had similar expression trends with transcriptome analysis results, with higher expression in red-skinned “Starkrimson” than its green variant (Figure 5). This indicated that these genes were related to the anthocyanin biosynthesis and regulated the formation of red skinned pear. Meanwhile, *LAR* (Pbr027485.1) and *ANR* (Pbr000218.1) were more highly expressed in the green variant than red “Starkrimson” (Figure 5), which promote the process of PA pathway and contributed to the formation of green skinned pear. In addition, the trends of the genes Pbr008092.1 and Pbr021636.1 were consistent with anthocyanin biosynthesis and accumulation in fruit developmental stages, while the genes of Pbr019417.1 and Pbr041849.1 showed opposing changes. qRT-PCR further demonstrated that genes related to metabolism of cofactors and vitamins (Pbr019417.1), amino acid metabolism (Pbr036011.1, Pbr041919.1, Pbr012649.1), carbohydrate metabolism (Pbr008092.1), genetic information processing (Pbr041849.1), and other regulated gene (Pbr036167.1) showed significant difference between red-skinned pear “Starkrimson” and its green mutant, indicating that anthocyanin accumulation in pear fruit is related to many biological processes.

## Conclusions

The present results demonstrate the usefulness of the digital transcript abundance measurement approach for identifying critical DEGs in the red-skinned pear “Starkrimson” and its green mutant. A list of candidate genes for functional studies involving anthocyanin/ proanthocyanidin biosynthesis and regulations was generated, among which *LAR* and *ANR* appeared to play a key role in promoting the proanthocyanin biosynthesis pathway. Their expression led to low anthocyanin accumulation in fruit skin, showing that *LAR* and *ANR* are associated with the red/green skin color mutant of pear. Furthermore, several transcription factors, *MYB, AP2, WRKY*, and *MADS*, were identified as differentially expressed in red/green color mutant, indicating that multiple transcription factors are involved in the regulation of anthocyanin/proanthocyanidin biosynthesis in pear fruit and lead to different fruit colors. The qRT-PCR results also indicated that the screening candidate genes *AP2* and *WARK* were involved in red skinned pear formation, and *LAR* and *ANR* were related to the development of green skin. Further studies should concentrate on functional characterization of these genes. This study could lead to better understanding of the global network of differentially expressed genes for red or green coloration in pear fruits.

### Conflict of interest statement

The authors declare that the research was conducted in the absence of any commercial or financial relationships that could be construed as a potential conflict of interest.

## Supplementary material

Additional File 1 **MYB-binding cis-motifs in the promoter region of the PA pathway genes ANR and LAR in pears**.

## References

[B1] Abrahams S. Lee E. Walker A. R. Tanner G. J. Larkin P. J. Ashton A. R. (2003). The Arabidopsis TDS4 gene encodes leucoanthocyanidin dioxygenase (LDOX) and is essential for proanthocyanidin synthesis and vacuole development. Plant J. 35, 624–636. 10.1046/j.1365-313X.2003.01834.x 12940955

[B2] Aharoni A. De Vos C. H. Wein M. Sun Z. Greco R. Kroon A.. (2001). The strawberry *FaMYB1* transcription factor suppresses anthocyanin and flavonol accumulation in transgenic tobacco. Plant J, 28, 319–332. 10.1046/j.1365-313X.2001.01154.x 11722774

[B3] Akagi T. Ikegami A. Tsujimoto T. Kobayashi S. Sato A. Kono A.. (2009). DkMyb4 is a Myb transcription factor involved in proanthocyanidin biosynthesis in Persimmon fruit. Plant Physiol. 151, 2028–2045. 10.1104/pp.109.146985 19783643 PMC2785967

[B4] Alfenito M. R. Souer E. Goodman C. D. Buell R. Mol J. Koes R.. (1998). Functional complementation of anthocyanin sequestration in the vacuole by widely divergent glutathione S-transferases. Plant Cell 10, 1135–1149. 9668133 10.1105/tpc.10.7.1135 PMC144053

[B5] Ashburner M. Ball C. A. Blake J. A. Botstein D. Butler H. Cherry J. M.. (2000). Gene Ontology: tool for the unification of biology. Nat. Genet. 25, 25–29. 10.1038/75556 10802651 PMC3037419

[B6] Audic S. Claverie J. M. (1997). The significance of digital gene expression profiles. Genome Res. 7, 986–995. 9331369 10.1101/gr.7.10.986

[B7] Ban Y. Honda C. Hatsuyama Y. Igarashi M. Bessho H. Moriguchi T. (2007). Isolation and functional analysis of a MYB transcription factor gene that is a key regulator for the development of red coloration in apple skin. Plant Cell Physiol. 48, 958–970. 10.1093/pcp/pcm066 17526919

[B8] Benjamini Y. Yekutieli D. (2001). The control of the false discovery rate in multiple testing under dependency. Ann. Stat. 29, 1165–1188.

[B9] Bieza K. Lois R. (2001). An Arabidopsis mutant tolerant to lethal ultraviolet-B levels shows constitutively elevated accumulation of flavonoids and other phenolics. Plant Physiol. 126, 1105–1115. 10.1104/pp.126.3.1105 11457961 PMC116467

[B10] Borevitz J. O. Xia Y. Blount J. Dixon R. A. Lam C. (2000). Activation tagging identifies a conserved MYB regulator of phenylpropanoid biosynthesis. Plant Cell 12, 2383–2393. 10.1105/tpc.12.12.2383 11148285 PMC102225

[B11] Chagné D. Gasic K. Crowhurst R. N. Han Y. Bassett H. C. Bowatte D. R.. (2008). Development of a set of SNP markers present in expressed genes of the apple. Genomics 92, 353–358. 10.1016/j.ygeno.2008.07.008 18721872

[B12] Cheng C. X. Jiao C. Singer S. D. Gao M. Xu X. Z. Zhou Y. M.. (2015). Gibberellin-induced changes in the transcriptome of grapevine (*Vitis labrusca* × *V. vinifera)* cv. Kyoho flowers. BMC Genomics 16:128. 10.1186/s12864-015-1324-8 25888129 PMC4348105

[B13] Conesa A. Götz S. García-Gómez J. M. Terol J. Talón M. Robles M. (2005). Blast2GO: a universal tool for annotation, visualization and analysis in functional genomics research. Bioinformatics 21, 3674–3676. 10.1093/bioinformatics/bti610 16081474

[B14] Conn S. Curtin C. Bézier A. Franco C. Zhang W. (2008). Purification, molecular cloning, and characterization of glutathione S-transferases (GSTs) from pigmented *Vitis vinifera* L. cell suspension cultures as putative anthocyanin transport proteins. J. Exp. Bot. 59, 3621–3634. 10.1093/jxb/ern217 18836188 PMC2561157

[B15] Dardick C. D. Callahan A. M. Chiozzotto R. Schaffer R. J. Piagnani M. C. Scorza R. (2010). Stone formation in peach fruit exhibits spatial coordination of the lignin and flavonoid pathways and similarity to Arabidopsis dehiscence. BMC Biol. 8:13. 10.1186/1741-7007-8-13 20144217 PMC2830173

[B16] Davies K. M. Schwinn K. E. (2003). Transcriptional regulation of secondary metabolism. Funct. Plant Biol. 30, 913–925. 10.1071/FP03062 32689076

[B17] Espley R. V. Hellens R. P. Putterill J. Stevenson D. E. Kutty-Amma S. Allan A. C. (2007). Red colouration in apple fruit is due to the activity of the MYB transcription factor, *MdMYB10*. Plant J. 49, 414–427. 10.1111/j.1365-313X.2006.02964.x 17181777 PMC1865000

[B18] Feng S. Wang Y. Yang S. Xu Y. Chen X. (2010). Anthocyanin biosynthesis in pears is regulated by a R2R3-MYB transcription factor *PyMYB10*. Planta 232, 245–255. 10.1007/s00425-010-1170-5 20422209

[B19] Fischer T. C. Gosch C. Pfeiffer J. Halbwirth H. Halle C. Stich K. (2007). Flavonoid genes of pear (*Pyrus communis*). Trees 21, 521–529. 10.1007/s00468-007-0145-z

[B20] Fischer T. C. Mirbeth B. Rentsch J. Sutter C. Ring L. Flachowsky H.. (2014). Premature and ectopic anthocyanin formation by silencing of anthocyanidin reductase in strawberry (*Fragaria* × *ananassa*). New Phytol. 201, 440–451. 10.1111/nph.12528 24117941

[B21] Gonzalez A. Zhao M. Leavitt J. M. Lloyd A. M. (2008). Regulation of the anthocyanin biosynthetic pathway by the TTG1/bHLH/Myb transcriptional complex in Arabidopsis seedlings. Plant J. 53, 814–827. 10.1111/j.1365-313X.2007.03373.x 18036197

[B22] Grabherr M. G. Haas B. J. Yassour M. Levin J. Z. Thompson D. A. Amit I.. (2011). Full-length transcriptome assembly from RNA-Seq data without a reference genome. Nat. Biotechnol. 29, 644–652. 10.1038/nbt.1883 21572440 PMC3571712

[B23] Griesser M. Vitzthum F. Fink B. Bellido M. L. Raasch C. Munoz-Blanco J.. (2008). Multi-substrate flavonol O-glucosyltransferases from strawberry (*Fragariaxananassa*) achene and receptacle. J. Exp. Bot. 59, 2611–2625. 10.1093/jxb/ern117 18487633 PMC2486459

[B24] Guo F. Yu H. W. Xu Q. Deng X. (2015). Transcriptomic analysis of differentially expressed genes in an orange-pericarp mutant and wild type in pummelo (*Citrus grandis*). BMC Plant Biol. 15:44. 10.1186/s12870-015-0435-3 25849782 PMC4352283

[B25] Hichri I. Heppel S. C. Pillet J. Léon C. Czemmel S. Delrot S.. (2010). The basic helix-loop-helix transcription factor *MYC1* is involved in the regulation of the flavonoid biosynthesis pathway in grapevine. Mol. Plant 3, 509–523. 10.1093/mp/ssp118 20118183

[B26] Holton T. A. Cornish E. C. (1995). Genetics and biochemistry of anthocyanin biosynthesis. Plant Cell. 7:1071. 10.1105/tpc.7.7.1071 12242398 PMC160913

[B27] Jain M. Ghanashyam C. Bhattacharjee A. (2010). Comprehensive expression analysis suggests overlapping and specific roles of rice glutathione S-transferase genes during development and stress responses. BMC Genomics 11:73. 10.1186/1471-2164-11-73 20109239 PMC2825235

[B28] Kanehisa M. Araki M. Goto S. Hattori M. Hirakawa M. Itoh M.. (2008). KEGG for linking genomes to life and the environment. Nucl. Acids Res. 36, D480–D484. 10.1093/nar/gkm882 18077471 PMC2238879

[B29] Kanehisa M. Goto S. Hattori M. Aoki-Kinoshita K. F. Itoh M. Kawashima S.. (2006). From genomics to chemical genomics: new developments in KEGG. Nucleic Acids Res. 34, D354–D357. 10.1093/nar/gkj102 16381885 PMC1347464

[B30] Kitamura S. Shikazono N. Tanaka A. (2004). TRANSPARENT TESTA 19 is involved in the accumulation of both anthocyanins and proanthocyanidins in Arabidopsis. Plant J. 37, 104–114. 10.1046/j.1365-313X.2003.01943.x 14675436

[B31] Konczak I. Zhang W. (2004). Anthocyanins-more than nature's colours. J. Biomed. Biotechnol. 5, 239–240. 10.1155/S1110724304407013 15577183 PMC1082903

[B32] Larsen E. S. Alfenito M. R. Briggs W. R. Walbot V. (2003). A carnation anthocyanin mutant is complemented by the glutathione S-transferases encoded by maize *Bz2* and petunia *An9*. Plant Cell Rep. 21, 900–904. 10.1007/s00299-002-0545-x 12789508

[B33] Lee H. S. Wicher L. (1991). Anthocyanin pigments in the skin of lychee fruit. J. Food Sci. 56, 466 10.1111/j.1365-2621.1991.tb05305.x

[B34] Lin-Wang K. Bolitho K. Grafton K. Kortstee A. Karunairetnam S. McGhie T. K.. (2010). An R2R3 MYB transcription factor associated with regulation of the anthocyanin biosynthetic pathway in Rosaceae. BMC Plant Biol. 10:50. 10.1186/1471-2229-10-50 20302676 PMC2923524

[B35] Lin-Wang K. Micheletti D. Palmer J. Volz R. Lozano L. Espley R.. (2011). High temperature reduces apple fruit colour via modulation of the anthocyanin regulatory complex. Plant Cell Environ. 34, 1176–1190. 10.1111/j.1365-3040.2011.02316.x 21410713

[B36] Liu P. Xue C. Wu T. T. Heng W. Jia B. Ye Z. F.. (2013a). Molecular analysis of the processes of surface brown spot (SBS) formation in pear fruit (*Pyrus bretschneideri Rehd*. cv. Dangshansuli) by de novo transcriptome assembly. PLoS ONE 9:e74217. 10.1371/journal.pone.0074217 24058529 PMC3776823

[B37] Liu X. F. Feng C. Zhang M. M. Yin X. R. Xu C. J. Chen K. S. (2013c). The *MrWD40-1* gene of chinese Bayberry (*Myrica rubra*) interacts with MYB and bHLH to enhance anthocyanin accumulation. Plant Mol. Biol. Rep. 31, 1474–1484. 10.1007/s11105-013-0621-0

[B38] Liu Y. Shi Z. Maximova S. Payne M. J. Guiltinan M. J. (2013b). Proanthocyanidin synthesis in Theobroma cacao: genes encoding anthocyanidin synthase, anthocyanidin reductase, and leucoanthocyanidin reductase. BMC Plant Biol. 13:202. 10.1186/1471-2229-13-202 24308601 PMC4233638

[B39] Marrs K. A. Alfenito M. R. Lloyd A. M. Walbot V. (1995). glutathione S-transferase involved in vacuolar transfer encoded by the maize gene Bronze-2. Nature 375, 397–400. 7760932 10.1038/375397a0

[B40] Matsui K. Umemura Y. Ohme-Takagi M. (2008). *AtMYBL2*, a protein with a single MYB domain, acts as a negative regulator of anthocyanin biosynthesis in Arabidopsis. Plant J. 55, 954–967. 10.1111/j.1365-313X.2008.03565.x 18532977

[B41] Medina-Puche L. Cumplido-Laso G. Amil-Ruiz F. Hoffmann T. Ring L. Rodríguez-Franco A.. (2014). *MYB10* plays a major role in the regulation of flavonoid/phenylpropanoid metabolism during ripening of Fragaria ananassa fruits. J. Exp. Bot. 65, 401–417. 10.1093/jxb/ert377 24277278

[B42] Mortazavi A. Williams B. A. McCue K. Schaeffer L. Wold B. (2008). Mapping and quantifying mammalian transcriptomes by RNA-Seq. Nature Methods 5, 621–628. 10.1038/nmeth.1226 18516045 PMC13303166

[B43] Pan F. G. Zhao Y. Y. Zhu S. Sun C. J. Lei L. C. Feng X.. (2012). Different transcriptional profiles of RAW264.7 infected with *mycobacterium tuberculosis* H37Rv and *BCG* identified via deep sequencing. PLoS ONE 7:e51988. 10.1371/journal.pone.0051988 23284841 PMC3526534

[B44] Pertea G. Huang X. Liang F. Antonescu V. Sultana R. Karamycheva S.. (2003). *TIGR* Gene Indices clustering tools (TGICL): a software system for fast clustering of large EST datasets. Bioinformatics 19, 651–652. 10.1093/bioinformatics/btg034 12651724

[B45] Pierantoni L. Dondini L. De Franceschi P. Musacchi S. Winke B. S. Sansavini S. (2010). Mapping of an anthocyanin-regulating MYB transcription factor and its expression in red and green pear, *Pyrus communis*. Plant Physiol. Biochem. 48, 1020–1026. 10.1016/j.plaphy.2010.09.002 20951056

[B46] Qi X. Wu J. Wang L. Li L. Cao Y. Tian L.. (2013). Identifying the candidate genes involved in the calyx abscission process of “Kuerlexiangli” (*Pyrus sinkiangensis* Yu) by digital transcript abundance measurements. BMC Genomics 14:727. 10.1186/1471-2164-14-727 24152304 PMC4046677

[B47] Rahim M. A. Busatto N. Trainotti L. (2014). Regulation of anthocyanin biosynthesis in peach fruits. Planta 240, 913–929. 10.1007/s00425-014-2078-2 24827911

[B48] Ramsay N. A. Glover B. J. (2005). MYB–bHLH–WD40 protein complex and the evolution of cellular diversity. Trends Plant Sci. 10, 63–70. 10.1016/j.tplants.2004.12.011 15708343

[B49] Rowan D. D. Cao M. Lin-Wang K. Cooney J. M. Jensen D. J. Austin P. T.. (2009). Environmental regulation of leaf colour in red *35S:PAP1* Arabidopsis thaliana. New Phytol. 182, 102–115. 10.1111/j.1469-8137.2008.02737.x 19192188

[B50] Schijlen E. G. Ric de Vos C. H. van Tunen J. Bovy A. G. (2004). Modification of flavonoid biosynthesis in crop plants. Phytochemistry 65, 2631–2648. 10.1016/j.phytochem.2004.07.028 15464151

[B51] Shi S. G. Yang M. Zhang M. Wang P. Kang Y. X. Liu J. J. (2014). Genome-wide transcriptome analysis of genes involved in flavonoid biosynthesis between red and white strains of Magnolia sprengeri pamp. BMC Genomics 15:706. 10.1186/1471-2164-15-706 25150046 PMC4156625

[B52] Takos A. M. Jaffé F. W. Jacob S. R. Bogs J. Robinson S. P. Walker A. R. (2006). Light-induced expression of a MYB gene regulates anthocyanin biosynthesis in red apples. Plant Physiol. 142, 1216–1232. 10.1104/pp.106.088104 17012405 PMC1630764

[B53] Tanaka Y. Sasaki N. Ohmiya A. (2008). Biosynthesis of plant pigments: anthocyanins, betalains and carotenoids. Plant J. 54, 733–749. 10.1111/j.1365-313X.2008.03447.x 18476875

[B54] Tanner G. J. Francki K. T. Abrahams S. Watson J. M. Larkin P. J. Ashton A. R. (2003). Proanthocyanidin biosynthesis in plants: purification of legume leucoanthocyanidin reductase and molecular cloning of its cDNA. J. Biol. Chem. 278, 31647–31656. 10.1074/jbc.M302783200 12788945

[B55] Telias A. Lin-Wang K. Stevenson D. E. Cooney J. M. Hellens R. P. Allan A. C.. (2011). Apple skin patterning is associated with differential expression of *MYB10*. BMC Plant Biol. 11:93. 10.1186/1471-2229-11-93 21599973 PMC3127826

[B56] Veeriah S. Kautenburger T. Habermann N. Sauer J. Dietrich H. Will F.. (2006). Apple flavonoids inhibit growth of HT29 human colon cancer cells and modulate expression of genes involved in the biotransformation of xenobiotics. Mol. Carcinog. 45, 164–174. 10.1002/mc.20158 16369997

[B57] Wang Y. Z. Dai M. S. Zhang S. J. Shi Z. B. (2014). Exploring candidate genes for pericarp russet pigmentation of sand pear (*Pyrus pyrifolia*) via RNA-Seq data in two genotypes contrasting for pericarp color. PLoS ONE 9:e83675. 10.1371/journal.pone.0083675 24400075 PMC3882208

[B58] Wang Z. Meng D. Wang A. Li T. Jiang S. Cong P.. (2013). The Methylation of the *PcMYB10* promoter is associated with green-skinned sport in Max Red Bartlett Pear. Plant Physiol. 162, 885–896. 10.1104/pp.113.214700 23629835 PMC3668077

[B59] Wu J. Wang Z. Shi Z. Zhang S. Ming R. Zhu S.. (2013a). The genome of the pear (*Pyrus bretschneideri Rehd*.). Genome Res. 23, 396–408. 10.1101/gr.144311.112 23149293 PMC3561880

[B60] Wu J. Zhao G. Yang Y. N. Yue W. Q. Khan M. A. Zhang S. L. (2013b). Identification of differentially expressed genes related to coloration in red/green mutant pear (*Pyrus communis L*.). Tree Genet. Genomes 9, 75–83. 10.1007/s11295-012-0534-3

[B61] Wu T. Zhang R. Gu C. Wu J. Wan H. Zhang S. (2012). Evaluation of candidate reference genes for real time quantitative PCR normalization in pear fruit. Afr. J. Agr. Res. 7, 3701–3704. 10.5897/AJAR11.1842

[B62] Xie D. Y. Sharma S. B. Paiva N. L. Ferreira D. Dixon R. A. (2003). Role of anthocyanidin reductase, encoded by BANYULS in plant flavonoid biosynthesis. Science 299, 396–399. 10.1126/science.1078540 12532018

[B63] Xie X. B. Shen L. S. Zhang R. F. Zhao J. Chen Y. C. Zhao Q.. (2012). The *bHLH* transcription factor *MdbHLH3* promotes anthocyanin accumulation and fruit coloration in response to low temperature in apples. Plant Cell Environ. 35, 1884–1897. 10.1111/j.1365-3040.2012.02523.x 22519753

[B64] Xue Y. Zhang Y. Grace S. He Q. F. (2014). Functional expression of an Arabidopsis p450 enzyme, p -coumarate-3-hydroxylase, in the cyanobacterium synechocystis PCC 6803 for the biosynthesis of caffeic acid. J. Appl. Phycol. 26, 219–226. 10.1007/s10811-013-0113-5

[B65] Yamagishi M. Shimoyamada Y. Nakatsuka T. Masuda K. (2010). Two R2R3-MYB genes, homologs of Petunia *AN2*, regulate anthocyanin biosyntheses in flower tepals, tepal spots and leaves of asiatic hybrid lily. Plant Cell Physiol. 51, 463–474. 10.1093/pcp/pcq011 20118109

[B66] Yang Y. N. Yao G. F. Zheng D. Zhang S. L. Wang C. Zhang M. Y. (2015). Expression differences of anthocyanin biosynthesis genes reveal regulation patterns for red pear coloration. Plant Cell Rep. 34, 189–198. 10.1007/s00299-014-1698-0 25323492

[B67] Yang Y. N. Zhao G. Yue W. Q. Zhang S. L. Gu C. Wu J. (2013). Molecular cloning and gene expression differences of the anthocyanin biosynthesis-related genes in the red/green skin color mutant of pear (*Pyrus communis* L.). Tree Genet. Genomes 9, 1351–1360. 10.1007/s11295-013-0644-6

[B68] Yu B. Zhang D. Huang C. H. Qian M. J. Zheng X. Y. Teng Y. W. (2012). Isolation of anthocyanin biosynthetic genes in red Chinese sand pear (*Pyrus pyrifolia Nakai*) and their expression as affected by organ/tissue, cultivar, bagging and fruit side. Sci. Horticult. 136, 29–37. 10.1016/j.scienta.2011.12.026

[B69] Zhang B. Hu Z. Zhang Y. Li Y. Zhou S. Chen G. (2012). A putative functional MYB transcription factor induced by low temperature regulates anthocyanin biosynthesis in purple kale (*Brassica oleracea ar. acephala f. tricolor*). Plant Cell Rep. 31, 281–289. 10.1007/s00299-011-1162-3 21987119

[B70] Zhang F. Gonzalez A. Zhao M. Payne C. T. Lloyd A. (2003). A network of redundant bHLH proteins functions in all TTG1- dependent pathways of Arabidopsis. Development 130, 4859–4869. 10.1242/dev.00681 12917293

[B71] Zhang Q. J. Tao S. T. Li M. Qi X. X. Wu J. Yin H. (2015). Identification of differentially expressed genes using digital gene expression profiles in *Pyrus pyrifolia Nakai* cv. Hosui bud release following early defoliation. Tree Genet. Genomes 11:34 10.1007/s11295-015-0858-x

[B72] Zhang X. Allan A. C. Yi Q. Chen L. Li K. Shu Q. (2011). Differential gene expression analysis of Yunnan Red Pear, *Pyrus Pyrifolia*, during fruit skin coloration. Plant Mol. Biol. Rep. 29, 305–314. 10.1007/s11105-010-0231-z

[B73] Zhou H. Lin-Wang K. Wang H. Gu C. Dare A. P. Espley R. V.. (2015). Molecular genetics of blood-fleshed peach reveals activation of anthocyanin biosynthesis by NAC transcription factors. Plant J. 82, 105–121. 10.1111/tpj.12792 25688923

[B74] Zhou Y. Zhou H. Lin-Wang K. Vimolmangkang S. Espley R. V. Wang L.. (2014). Transcriptome analysis and transient transformation suggest an ancient duplicated MYB transcription factor as a candidate gene for leaf red coloration in peach. BMC Plant Biol. 14:388. 10.1186/s12870-014-0388-y 25551393 PMC4302523

