# Supplementary figures and images for: Transcriptome profiling reveals differential gene expression in proanthocyanidin biosynthesis associated with red/green skin color mutant of pear (Pyrus communis L.)
Source: Front Plant Sci. 2015 Sep 30;6:795. doi: 10.3389/fpls.2015.00795 (PMC4588701; doi:10.3389/fpls.2015.00795)

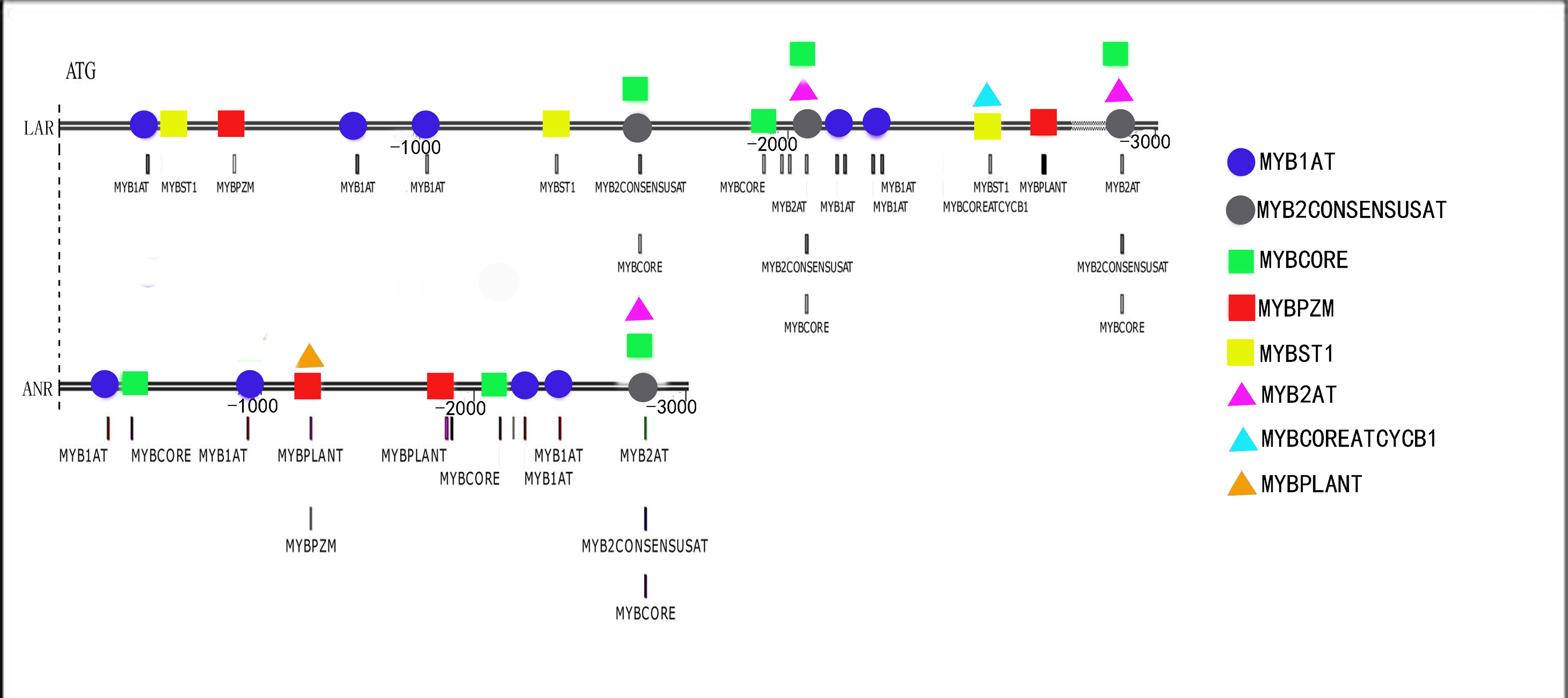

Supplement: Additional File 1 — MYB-binding cis-motifs in the promoter region of the PA pathway genes ANR and LAR in pears. [file Image1.JPEG]
